# Studies of Focal Infections about the Teeth*Read before Toronto Dental Society, November, 1917, and reprinted from “Oral Health” for February. This article raises several important questions that require careful study, and it suggests material improvement in our treatment of this condition.

**Published:** 1918-03

**Authors:** Arthur D. Black

**Affiliations:** Chicago


### STUDIES OF FOCAL INFECTIONS ABOUT THE TEETH*

BY ARTHUR D. BLACK, A.M., M.D., D.D.S., CHICAGO

In considering the chronic infections of the mouth—alveolar abscess and suppurative pericementitis—we should have in mind the relationship of the enamel and the gingivae to the deeper tissues. Probably we all appreciate the protective function of the enamel in relation to the pulp, and therefore in relation to the occurrence of alveolar abscess. So long as the enamel is kept intact by prophylactic measures, or decayed areas are discovered while yet small and replaced by fillings which are not too deep, the pulp will, as a rule, remain vital and abscess will be prevented. We should appreciate the fact that the gingivae perform practically the same protective function for the peridental membranes. So long as the gingivae are maintained in a state of health by proper care by the patient and due consideration by the dentist, disease of the peridental membrane will not occur. The gingivae

* Read before Toronto Dental Society, November, 1917, and reprinted from "Oral Health" for February. This article raises several important questions that require careful study, and it suggests material improvement in our treatment of this condition.

are so constituted and so supplied with blood that they are enabled to withstand much of irritation and injury without breaking down. Probably no tissue heals more promptly when injured. Thus they protect the deeper tissues, which have not the same powers of repair.

In presenting this subject I will presume that the relationship of these chronic mouth foci to the general health is recognized. This being so, we have only to look at certain statistics which have been collected to appreciate the very serious problem which confronts the dental profession. During the past few years I have endeavored to collect data as to the incidence of these two types of chronic mouth infections. This data has been gathered by roentgenographic examination of the bone adjacent to the teeth. At the outset it was realized that most roentgenographs are taken because there is some indication for doing so. I therefore undertook the roentgenographic examinations of mouths of persons without regard to the condition of their mouths or health. A first tabulation was presented to the American Medical Association † last June.

For this paper I have copied selected parts of the tabulation, which covered the examination of three hundred mouths, and the percentage of persons of different ages having alveolar abscesses or pus pockets along the sides of the roots are given. It will be noted that practically half of all persons twenty-five years of age have one or more chronic alveolar abscesses and that the percentage gradually increases up to the age of fifty, when it becomes less, apparently because of extractions.

Cases of chronic pericementitis have their beginning in the mouths of adults, there being few cases before the age of twenty-five. There is a rapid increase from nine per cent. to thirty-two per cent. between the ages of twenty-five and thirty, and a further increase to sixty-four per cent. at forty years, continuing to seventy-four per cent. at fifty, and ninety-two per cent. in the mouths of those examined who were over fifty. It should be noted that in this tabulation there were included a number of persons over forty who presented because of

| Age | Number examined | Average number of teeth per person | Percentage of persons, bone destroyed about apices of roots | Percentage of persons, bone destroyed at sides of roots | Percentage having one or both forms of infection of maxillary bones | Total number of teeth with root fillings | Number having good root fillings | Number abscessed with good root fillings | Number having poor root fillings | Number abscessed with poor root filling | Number root fillings not considered because not clearly shown |
|---|---|---|---|---|---|---|---|---|---|---|---|
| 17 to 25......... | 86 | 30 | 52 | 9 | 56 | 198 | 79 | 3 | 143 | 101 | 35 |
| 25 to 29......... | 53 | 29 | 53 | 32 | 72 | 121 | 50 | 10 | 79 | 54 | 18 |
| 30 to 39......... | 68 | 26 | 66 | 64 | 87 | 236 | 59 | 5 | 159 | 89 | 50 |
| 40 to 49......... | 53 | 25 | 68 | 74 | 89 | 185 | 38 | 3 | 129 | 83 | 34 |
| 50 and over..... | 40 | 23 | 55 | 92 | 100 | 111 | 47 | 2 | 70 | 52 | 15 |
| Tl. or average... | 300 | 27 | 59 | 51 | 77 | 851 | 273 | 23 | 580 | 379 | 152 |

Total number of root fillings classified as good or poor, each root of multiple rooted teeth being counted separately.................................. 853

Number of above having good root fillings, 273; number of these abscessed, 23, which is.......................................................... 8%

Number of above having poor root fillings, 580; number of these abscessed, 379, which is.......................................................... 65%

physical disability and the figures are therefore somewhat higher than the average for persons over forty years of age.

The number of teeth was counted for each person and it is interesting to note the gradual reduction with advancing years. The fact that the number of teeth per individual is so high for this group—thirty for those under twenty-five and twenty-three for those over fifty—is an indication that the persons examined were probably considerably above the general average in the care of their mouths.

There has been much discussion of the occurrence of abscesses following pulp removal and root filling. Some men have expressed the belief that all pulpless teeth should be extracted. In this tabulation two things stand out very clearly; first, the percentage of abscesses occurring when the roots are well filled is small; second, the percentage of poor root fillings is very high, indicating much lack of care in technic, and the percentage of abscesses resulting from poor root fillings is very large—sixty-seven per cent. In estimating the percentage of abscesses is due to destruction of the tissues about the teeth no allowance was made for teeth which were abscessed before the root fillings were made, the records of the previous condition and of the treatment of the teeth not being available. These figures should carry home to each of us the importance of greater care in pulp treatment and root filling. It should also give those whose technic is thorough and painstaking a feeling of confidence for the future.

Attention is called to the fact that we have very little accurate data as to the causes of chronic alveolar abscess. We know that abscess occurs as the result of the death of the pulp, but no estimate has been made of the percentages which occur in cases in which the pulp was vital when treatment was begun. It is likely that a percentage of abscesses are due to destruction of the tissues about the apex by arsenic, that many are caused by medicaments sealed in the canals as "dressings," many are doubtless caused by faulty technic in pulp removal or root filling. It is not within the scope of this paper to discuss these questions. I simply wish to impress the fact that we can not have accurate knowledge on these matters until a considerable number of men keep and publish accurate records of cases, noting the condition of each pulp when treatment was undertaken, the various medicaments used, etc., and the final result as shown by roentgenographs taken a year or so after making the root filling. When this information is available, we will doubtless modify present plans of treatment. Until such time as the facts are before us, I think we will do well not to use drugs which will destroy soft tissue by contact, as there can be little assurance that some of the medicament sealed in the canal will not penetrate the apical foramen and reach the tissues beyond.

The percentage of adults presenting with suppurative processes involving the investing tissues of the teeth, as shown by the table, should impress upon every dentist the seriousness of the problem confronting us. If we are to do our duty in co-operation with the physician, we must not only eliminate these areas from the mouths of our patients, but also do what is much more important, prevent them in the largest possible measure in the future.

In the study of cases which may be thought to have resulted from focal infection, a word of caution should be given to both physicians and dentists. In view of the high percentage of mouth foci, it is probable that the search for other foci has been too frequently neglected. Since about seventy-five per cent. of all adults will be found to have either alveolar abscess or pus pockets along the sides of the roots, it follows that at least three out of every four persons presenting with secondary lesions, will be found to have infections involving the maxillary bones. It is feared that too often the search for original foci ends with the discovery of the mouth infection—and in many cases other and possibly more serious foci are overlooked, and there is no improvement following the elimination of the mouth lesions. In all cases the most thorough search should be made of all regions of primary infections. While the dentist may have done his duty in clearing the mouth of infection, it is proper that he should call to the attention of both patient and physician the need of the most thorough examination, which should include the nose and accessory sinuses, the tonsils, the middle ear, the genito-urinary tract, the gall bladder and the appendix. Tuberculosis, syphilis and gonorrhea are frequent causes of the secondary manifestations commonly resulting from focal infections.

As a basis for rational treatment if the mouth lesions, a study of the tissues and the pathological changes which occur is of the utmost importance. Our long delay in arriving at a proper understanding of the problem confronting us has been due in a large measure to a lack of application of our knowledge of the histological structure and physiological function of the various specialized elements which constitute the peridental membrane. The chronic suppuration which involves this tissue, whether in the form of an abscess at the apex of the root, or a detachment along the side of the root, destroys the connection of the peridental membrance between the root and the bone over a given area, and after a time the cementoblasts, which normally lie on the surface of the cementum, are destroyed, and the cementum is denuded. Subsequently the fibres of the peridental membrane disappear from the overlying soft tissue and the bone to which they were attached disappears, either by absorption or as a result of the suppuration, or both. I have cut away and examined microscopically many sections of this overlying tissue. It bears little or no resemblance to the former normal structure. All of the specialized elements are gone; it is no longer peridental membrane, and should not under any circumstances be expected to perform the functions of that tissue. The changes are comparable to those in other tissues containing specialized elements when involved by suppurations; the specialized elements are destroyed and are not rebuilt.

At the same time the cementum has become a dead tissue. It is practically a piece of necrosed bone, but the cementum lacks a circulation and has not the power possessed by bone, of exfoliating the dead portion. The denuded cementum therefore remains as a continuous irritant to the surrounding tissue and maintains the chronicity of these cases.

The man who recognizes these conditions must realize that reattachment of the overlying tissue does not occur; that the detachments are permanent. The treatment then at once becomes a simple matter. The root ends of abscessed teeth must be removed or the teeth must be extracted. The more I observe the cases in which root apices have been removed, the less I think of the operation. Extraction should be the standard and almost universal procedure.

For cases of chronic pericementitis in which there are pus pockets along the sides of the roots, we should think most of maintaining cleanliness and of measures to that end. While thorough scaling of teeth is important, it should be recognized that the deposits on the cementum are a result and not a cause of this condition and their removal must be considered only one item in the treatment. Wherever possible the overlying detached tissue should be cut away with the knife or cautery, leaving the denuded roots exposed. Shallow pockets may be cleaned twice daily by the patient, using a large rubber bulb syringe and normal saline solution. When the infection can not be controlled by these methods, the teeth should be extracted.

It was not the intention that this paper should take up the matter of treatment in detail. Its real object was to present, first, the high percentage of chronic infections involving the maxillary bones of adults; second, the pathological changes which occur in the peridental tissues—changes which are a natural result of chronic suppuration and which are fatal to these tissues so far as the possibilities of repair are concerned. These are simple facts which are promptly recognized by those who have seriously studied the tissues; facts which would make much of the treatment in vogue look ridiculous, if it were not for the very serious situation which confronts us as protectors of what is today the largest avenue of entrance of focal infections, and therefore the field of greatest menace to health.

